# Hypogelsolinemia, a disorder of the extracellular actin scavenger system, in patients with multiple sclerosis

**DOI:** 10.1186/1471-2377-10-107

**Published:** 2010-11-01

**Authors:** Alina Kułakowska, Nicholas J Ciccarelli, Qi Wen, Barbara Mroczko, Wiesław Drozdowski, Maciej Szmitkowski, Paul A Janmey, Robert Bucki

**Affiliations:** 1 Department of Neurology, Medical University of Białystok, 15-230 Białystok, Poland; 2 Institute for Medicine and Engineering, University of Pennsylvania, 1010 Vagelos Research Laboratories, 3340 Smith Walk, Philadelphia, PA, 19104 USA; 3 Department of Biochemical Diagnostics, Medical University of Białystok, 15-230 Białystok, Poland

## Abstract

**Background:**

Extracellular gelsolin (GSN) and GC-globulin/Vitamin D-binding protein (DBP) appear to play an important role in clearing the actin from extracellular fluids and in modulating cellular responses to anionic bioactive lipids. In this study we hypothesized that cellular actin release and/or increase in bioactive lipids associated with multiple sclerosis (MS) development will translate into alteration of the actin scavenger system protein concentrations in blood and cerebrospinal fluid (CSF) of patients with MS.

**Methods:**

We measured GSN and DBP concentrations in blood and CSF obtained from patients diagnosed with MS (n = 56) in comparison to a control group (n = 20) that includes patients diagnosed with conditions such as idiopathic cephalgia (n = 11), idiopathic (Bell's) facial nerve palsy (n = 7) and ischialgia due to discopathy (n = 2). GSN and DBP levels were measured by Western blot and ELISA, respectively.

**Results:**

We found that the GSN concentration in the blood of the MS group (115 ± 78 μg/ml) was significantly lower (p < 0.001) compared to the control group (244 ± 96 μg/ml). In contrast, there was no statistically significant difference between blood DBP concentrations in patients with MS (310 ± 68 μg/ml) and the control group (314 ± 82 μg/ml). GSN and DBP concentrations in CSF also did not significantly differ between those two groups.

**Conclusions:**

The decrease of GSN concentration in blood and CSF of MS subjects suggests that this protein may be involved in chronic inflammation associated with neurodegeneration. Additionally, the results presented here suggest the possible utility of GSN evaluation for diagnostic purposes. Reversing plasma GSN deficiency might represent a new strategy in MS treatment.

## Background

Actin binding is a common characteristic of both gelsolin and GC-globulin, also known as vitamin D binding protein. Gelsolin was first described as a ~90 kDa cytoplasmic actin-binding protein involved in the remodeling of cellular actin filaments associated with cell shape changes and movement [1]. Further studies revealed a secreted gelsolin isoform in the blood plasma [2,3]. As plasma gelsolin has the ability to bind actin, blood gelsolin depletion was observed in diverse states of inflammation associated with tissue injury and actin release: adult respiratory distress syndrome, sepsis, myocardial infarction, hepatitis, myonecrosis and rheumatoid arthritis [4-7]. In trauma patients, lower plasma gelsolin levels were associated with a poor prognosis [8]. Poor prognosis associated with lower plasma gelsolin levels was also shown in critically ill surgical patients [9] and in patients subjected to hemodialysis [10].

DBP acts as a carrier protein for vitamin D, but it can also be converted into a macrophage activating factor [11]. One of DBP's most important functions is to act as part of an extracellular actin scavenger system (EASS) [12,13]. This system is designed to remove actin from the blood plasma where it can form long filaments that can trigger intravascular coagulation and toxicity to endothelial cells [14,15]. In the actin scavenging system, gelsolin depolymerizes F-actin and DBP binds monomeric G-actin in order to prevent polymerization. This process allows actin to be cleared by the liver, but at the same time it consumes DBP and gelsolin [16,17]. Similar to gelsolin depletion, low DBP levels (both the total level and the actin-free level, which is an index of residual actin-scavenging capacity), was proposed to serve as a prognostic marker in situations of organ damage such as acute liver failure, and multiple trauma [18]. Other conditions such as septic shock may also be associated with reduced DBP levels and complex formation with actin [19]. In addition to actin, gelsolin and DBP bind important bioactive lipids. Gelsolin was shown to preferentially bind lysophosphatidic acid, platelet-activating factor, endotoxin, and lipoteichoic acid [20-22]. DBP specifically interacts with arachidonic acid, a precursor of prostaglandins [23]. Gelsolin's interaction with bioactive lipids can alter its ability to bind actin [24].

Multiple sclerosis (MS) is an immune-mediated demyelinating disorder of the human central nervous system. There is increasing evidence that axonal degeneration plays an important role in the pathogenesis of this disease. Some studies show that axonal cytoskeletal proteins such as actin, tubulin or the neurofilament light subunit (NFL) can be detected in CSF and blood from patients with neurodegenerative conditions and that the presence of these proteins might serve as useful confirmation of neurodegeneration or MS progression [25,26]. Analysis of gelsolin and DBP concentrations suggests the consideration of EASS protein analysis for diagnostic purposes. Additionally, hypogelsolinemia represents a potential therapeutic target for MS therapeutical intervention.

## Methods

### Specimen collections

Human blood and CSF specimen collection was performed in the Department of Neurology at the Medical University of Białystok in accordance with a protocol approved by the Medical University of Białystok Ethics Committee for Research on Humans and Animals, and written consent was obtained from all subjects. All individuals were already undergoing lumbar puncture for diagnostic purposes. Shortly after collection, samples of cerebrospinal fluid and blood were centrifuged (2000 × g, 20 min), and the supernatants of CSF and blood plasma were subjected to total protein analysis and frozen. The degree of neurological impairment in patients diagnosed with MS, from whom CSF was obtained, was evaluated using the Expanded Disability Status Scale (EDSS). All evaluations rated on average 1.5 ± 0.6, which indicates the early stages of MS. All MS patients suffered from the relapsing-remitting form of the disease and were not being treated with any disease-modifying drugs (beta-interferon, glatiramer acetate, natalizumab) or glucocorticosteroids at the time of hospital admission. Clinically, all MS patients included in the study represent category 1 or 2 according to the McDonald criteria [27]. All have a history of at least two neurological attacks (indicative of dissemination in time) and their single MRI shows T2-hyperdense lesions in different parts of the brain, but not all patients meet the Barkhof's MRI criteria of MS diagnosis [28]. Therefore, the lumbar puncture was performed to obtain a definitive diagnosis of MS. All CSF samples were clear, with the average number of lymphocytes equal to 4.1, and average Cl- and total protein concentrations of 132 ± 2.9 mEq/l and 381 μg/ml, respectively (Table 1). More importantly, oligoclonal bands type 2 or 3 were present in all CSF samples obtained from MS subjects, and QALB values indicate lack of blood/CSF barrier dysfunction in all subjects included in this study. We collected samples from 56 patients diagnosed with MS. However, due to some small CSF specimens, we were able to perform both gelsolin and DBP analysis in only 24 samples.

**Table 1:** Clinical and laboratory characteristics of the patient groups.

					CSF	
					
Clinical group	Total number (females)	Age (years)	EDSS	Q(Alb)	Total protein (μg/ml)	Lymphocytes
MS*	56 (30)	37.5 ± 10.5	1.5 ± 0.6	6.5 ± 1.1	381 ± 132	4.1 ± 2.3
Control:						
idiopathic cephalgia	11(9)	45.3 ± 25.3	N/A	6.9 ± 1.4	401 ± 165	3.1 ± 2.1
idiopathic (Bell's) facial nerve palsy	7(4)	52.2 ± 18.6	N/A	7.2 ± 0.8	360 ± 182	5.3 ± 2.2
ischialgia due to discopathy	2(0)	45.1 ± 26.2	N/A	6.8 ± 1.8	391 ± 192	4.2 ± 1.1

QAlb - albumin CSF/serum quotient. * oligoclonal IgG bands (type 2 or 3) were present in CSF of all subjects included in this group

### Immunoblotting of gelsolin in blood and CSF

Gelsolin concentration was assessed as previously described [29]. Briefly, after thawing, gel sample buffer was added to the plasma and CSF samples, which were then boiled and subjected to electrophoresis on 10% sodium dodecyl sulfate (SDS) - polyacrylamide minigels. Recombinant human plasma gelsolin was loaded as a positive control in each gel in a concentration range comparable to the gelsolin concentration in the samples (7.5 - 30 ng). After electrophoresis, proteins were transferred to PVDF membranes (Amersham, Biosciences Little Chalfont, UK), which were blocked by incubation in 5% (w/v) non-fat dry milk dissolved in TBS-T (150 mM NaCl, 50 mM TRIS, 0.05% Tween 20, pH = 7.4). Following transfer, proteins were probed with a monoclonal anti-human gelsolin antibody (G4896, Sigma, St. Louis, MO, USA) used at a 1:10,000 dilution in TBS-T. HRP-conjugated secondary antibodies were used at a 1:20,000 dilution in TBS-T. Immunoblots were developed with the Fuji Film LAS-300 system using an ECL Plus HRP-targeted chemiluminescent substrate (Amersham, Biosciences Little Chalfont, UK). The integrated intensity of each band on the Western Blot, minus the background signal was quantified with densitometrical analysis (Image Gauge - version 4.22 software; Fuji Photo Film Co, USA). All analyses were performed blind to clinical data.

### Determination of CG-globulin concentration

DBP was measured using a sandwich enzyme-linked immunosorbent assay (ELISA, BioPorto Diagnostics DK-2820 Gentofte, Denmark), according to the manufacturer's instructions.

### Identification of F-actin in CSF samples obtained from MS patients

F-actin presence in cerebrospinal fluid was evaluated using a technique previously used to detect F-actin in sera collected from patients with adult respiratory distress syndrome [15]. Briefly, CSF samples were subjected to centrifugation (13,500 × g, 4°C, for 20 min.), then 20 μl (1:200 dilution) of rhodamine (TRITC) phalloidin (Sigma, St Louis MO, USA) was added to the pellet. Samples were viewed in a Leica microscope (Bannockbum, IL, USA) using a 10× objective. Images were captured with a Cool SNAP(HQ) camera (Trenton, NJ, USA).

### Statistical analysis

Data are reported as mean ± SD from 20-40 specimens. Differences between means were evaluated using the unpaired Student's t-test, with p < 0.05 being taken as the level of significance. Additionally, values of gelsolin and DBP concentration in blood and CSF were compared using Pearson's correlation test.

## Results

### Gelsolin and vitamin D binding protein concentration in blood

**Figure 1:**
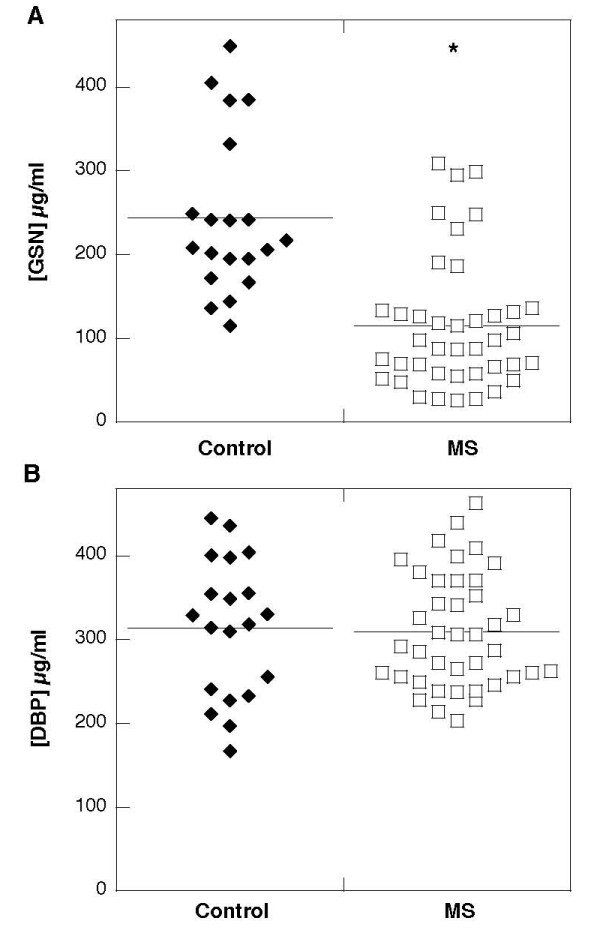
**Gelsolin (panel A) and Vitamin D binding protein (panel B) concentrations in blood collected from control patients (left) and subjects diagnosed with multiple sclerosis (right). **Horizontal bars depict means. *p < 0.001 compared to gelsolin concentration in control group.

Using quantitative western blot analysis we detected significantly lower (p < 0.001) gelsolin concentration in the blood of MS subjects (Figure 1) compared to the gelsolin concentration in our control group (115 ± 78 versus 244 ± 96 μg/ml). There was no significant difference between DBP levels in blood of MS patients and the control group (310 ± 68 μg/ml versus 314 ± 82), determined using ELISA. In the MS group, circulating gelsolin and DBP levels were similar in both males and females and were also not dependent on patient age. As all patients included in MS group were suffering from the relapsing-remitting form of the disease at early stages (Table 1), it may be concluded that depletion of plasma gelsolin represents an early event during MS development. Average gelsolin concentration in blood of MS patients was significantly lower (p < 0.001) compared to blood concentrations of vitamin D binding protein.

### Gelsolin and DBP concentration in cerebrospinal fluid

**Figure 2:**
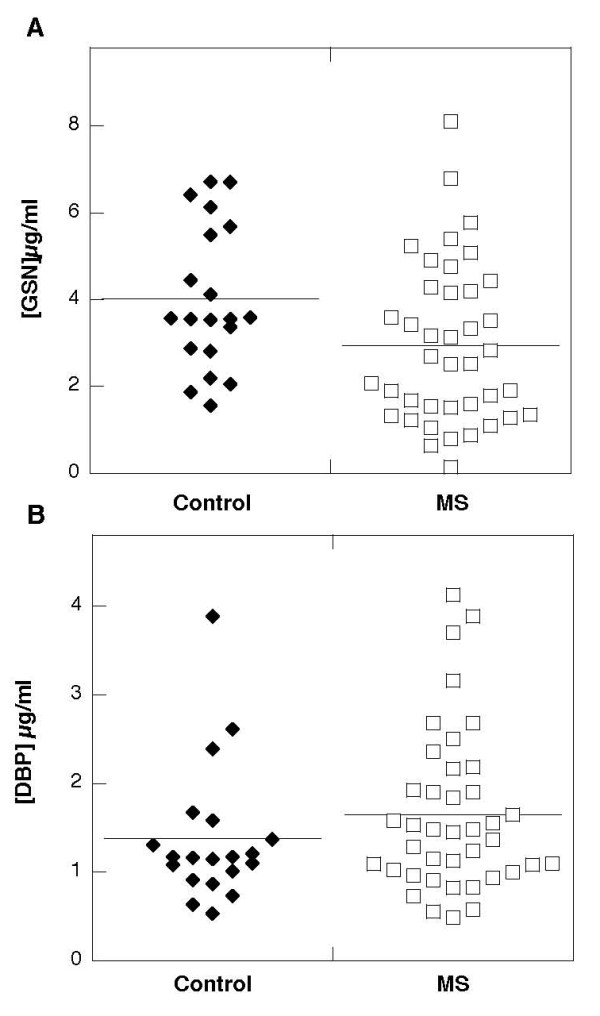
**Gelsolin (panel A) and Vitamin D binding protein (panel B) levels in cerebrospinal fluid collected from control patients (left) and subjects diagnosed with multiple sclerosis (right). **Horizontal bars depict means.

**Figure 3:**
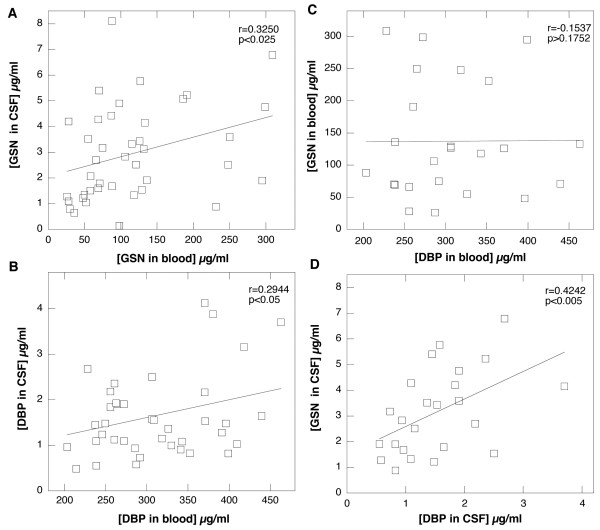
**Correlation between the blood and cerebrospinal fluid levels of gelsolin (panel A), the blood and cerebrospinal fluid levels of Vitamin D binding protein (panel B), blood gelsolin and blood DBP (panel C) and cerebrospinal fluid levels of DBP and gelsolin (panel D) in patients diagnosed with multiple sclerosis (MS). **Panels A/B and C/D include concentration values from 40 and 24 patients, respectively.

Our previous study showed that GSN present in CSF of MS patients consists predominantly of its plasma isoform, and using immunoblotting assays, we have not observed any cleavage products of pGSN in CSF [30]. Here we report that average gelsolin concentrations in CSF collected from patients diagnosed with MS was lower (2.9 ± 1.8 versus 4.1 ± 1.7 μg/ml) and the average DPB level was higher (1.6 ± 0.9 versus 1.4 ± 0.8 μg/ml) compared to the control group (Figure 2). The average gelsolin concentration in the CSF of MS patients was significantly higher (p < 0.001) than the concentration of vitamin D binding protein, showing that these plasma proteins do not simply partition passively into the CSF. Additionally, an analysis using Pearson's test indicated a weak correlation between gelsolin levels in CSF and its level in blood. A similar comparison for DBP also indicates a correlation between DBP concentrations in CSF and blood. However this correlation is weaker compared to that found for gelsolin levels in blood and CSF of MS subjects. The direction of gelsolin and DBP concentration changes in CSF (average values calculated based on 40 MS patients included in each group) suggests the possibility that depletion of CSF gelsolin levels might be compensated for by an increase in DBP concentration (Figure 2), but this statement is not supported by r (r = 0.4242) and p (p < 0.005) values calculated using Pearson's correlation test (it included 24 subjects in which GSN and DBP was analyzed in the same samples) indicating occurrence of positive correlation between those values in the same patients, potentially meaning that in subjects with higher CSF gelsolin concentration, higher DBP concentration is more likely to be observed (Figure 3).

### Presence of F-actin in CSF of MS subjects

**Figure 4:**
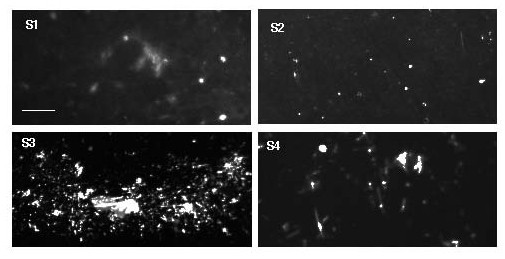
**Representative fluorescence images of F-actin in CSF pellets labeled with rhodamine - phalloidin. **Each panel (S1-S4) represents sample collected from different MS patients.Scale bars = 20 μm.

According to previous reports, F-actin is present in CSF of patients suffering from MS. However, we have not seen detectable amounts of actin in specimens of CSF collected from MS patients or some of our control group patients in Western blot analysis (data not shown). However, F-actin was detected in the pellets generated after centrifugation of ~250 μl of CSF samples followed by addition of rhodamine-phalloidin and microscopic observation (Figure 4).

## Discussion

This study confirms our previous report of a decrease of gelsolin concentration in the blood and CSF of a small group of MS patients (n = 4) [30]. For the current study, specimens were collected from 56 MS patients suffering from the relapsing - remitting form of the disease at early stages. Specimens were then subjected to analysis of the extracellular actin scavenger system proteins GSN and DBP. The results reveal significantly lower gelsolin concentration in blood of the MS group compared to the control group. However, lowered gelsolin concentration in CSF samples did not reach statistical significance when compared to its concentration in the control group. We have not observed any significant differences in DBP concentration in blood or CSF of MS subjects in comparison to the control, but the average DPB concentration in CSF from MS patients was marginally higher compared to the concentration in the control group.

The cause of observed EASS protein concentration changes in MS samples is unclear, but based on existing reports indicating higher levels of actin in CSF of MS patients [25], the presence of extravascular fibrinogen in MS lesions [31,32], dysfunction of the blood-brain barrier (BBB) associated with MS development, and the fact that the CNS itself becomes an immunological compartment with increasing cytokine production during the course of the disease [33], several possibilities can be considered. In the course of multiple sclerosis, axonal degeneration results in the release of actin and other cytoskeletal proteins within the intrathecal compartment, which may potentially mobilize blood EASS proteins to the CNS. Such mobilization would account for the decrease of gelsolin in blood, assuming that normal blood levels cannot be maintained by increasing production, which in the case of gelsolin occurs in muscle and neurons, but not liver. At the same time, higher demand for vitamin D binding protein will be filled more effectively by increased liver production of this acute phase reactant, which would explain the lack of significant changes in the DBP blood concentration. The identification of actin in the sediments of CSF obtained after centrifugation represents strong evidence to support this possibility. At the same time, these results are in agreement with the sequestration hypothesis that was proposed as a mechanism of actin-mediated gelsolin depletion [34]. The QAlb values indicate lack of CSF-barrier dysfunction in MS subjects included in our study, and it is very likely that the depletion of GSN and DBP from CSF that occurs as these proteins bind actin cannot be compensated for by an increased production or transport of gelsolin. Interestingly, in one recent study, up-regulation of gelsolin and DBP in CSF was reported for a pediatric MS group (n = 12) [35] suggesting that in the course of MS development, cerebrospinal fluid GSN and DBP concentrations may indeed increase in some stage of the disease. On the other hand, we observed a decrease of GSN in blood and CSF, which agrees with a study showing that a decrease of blood gelsolin level is inversely related to the intensity of systemic inflammation in patients with rheumatoid arthritis (RA) [7]. RA is a chronic inflammatory condition that shares similar pathophysiological processes with MS [36]. In addition, RA patients have significantly lower intra-articular plasma gelsolin levels in addition to the presence of actin-gelsolin complexes. On the other hand, in recent studies overexpression of gelsolin restored the impaired mitochondrial activity associated with CNS degeneration in the course of Alzheimer's disease (AD). Therefore, cytoplasmic gelsolin might act as a modulator of brain Abeta levels and its neurotoxic effects during AD development and may decrease neurodegeneration in AD [37].

Evaluation of MS biomarkers related to disease mechanisms, disease activity and progression, or therapeutic response is of great interest [38]. Our data give only a limited view of MS development and progression, and may be see as a preliminary result, so further long-term studies are required to evaluate the prognostic value of EASS protein level analysis as a biochemical marker for predicting and monitoring neurological decline in the course of MS. It is possible that EASS protein evaluation, especially gelsolin, in combination with other biomarker candidates such as neurofilament light chains, tau protein, actin, or tubulin, will increase diagnostic accuracy and better assessment of MS progression.

## Conclusion

The decrease of GSN concentration in blood and CSF of MS subjects indicates the involvement of this protein in the process of neurodegenerative disease development. Additionally, the results presented here suggest the possible utility of GSN evaluation for diagnostic purposes. Hypogelsolinemia represents a target for MS therapeutic intervention

## List of abbreviations

CSF: cerebrospinal fluid; DBP: GC-globulin/Vitamin D-binding protein; EDSS: Expanded Disability Status Scale; GSN: gelsolin; MS: multiple sclerosis; MRI: Magnetic resonance imaging; QAlb: albumin CSF/serum quotient; SDS: sodium dodecyl sulfate.

## Competing interests

In 2008, PAJ and RB were involved in a sponsored research agreement with Critical Biologics Inc. in a project directed at evaluating the potential clinical use of gelsolin, but not otherwise related to the present study. None of the research reported in this paper was supported by Critical Biologics Inc. or by any other corporate entity. The author's institution, University of Pennsylvania, has filed a patent application on which PAJ and RB are designated as inventors. The application concerns the diagnostic and therapeutic utility of gelsolin in neurological disorders. Other authors: none to declare.

## Authors' contributions

AK: participated in study design, collection of CSF samples, helped to draft the manuscript; NJC: carried out GSN studies; QW: statistical evaluation, helped to draft the manuscript; BM: carried out DBP studies helped to draft the manuscript; WD: participated in study design, helped to draft the manuscript; MS: participated in study design, helped to draft the manuscript; PAJ: participated in study design, helped to draft the manuscript; RB: carried out F-actin studies, participated in study design, helped to draft the manuscript. All authors read and approved the final manuscript.

## Pre-publication history

The pre-publication history for this paper can be accessed here:

http://www.biomedcentral.com/1471-2377/10/107/prepub
